# Fine-scale differences in eukaryotic communities inside and outside salmon aquaculture cages revealed by eDNA metabarcoding

**DOI:** 10.3389/fgene.2022.957251

**Published:** 2022-08-26

**Authors:** Marta Turon, Magnus Nygaard, Gledis Guri, Owen S. Wangensteen, Kim Præbel

**Affiliations:** ^1^ Norwegian College of Fishery Science, UiT The Arctic University of Norway, Tromsø, Norway; ^2^ Norwegian Institute of Marine Research, Tromsø, Norway

**Keywords:** eukaryotic communities, eDNA, aquaculture, metabarcoding, surface water, biodiversity, salmonid net pins, COI

## Abstract

Aquaculture impacts on marine benthic ecosystems are widely recognized and monitored. However, little is known about the community changes occurring in the water masses surrounding aquaculture sites. In the present study, we studied the eukaryotic communities inside and outside salmonid aquaculture cages through time to assess the community changes in the neighbouring waters of the farm. Water samples were taken biweekly over five months during the production phase from inside the cages and from nearby points located North and South of the salmon farm. Eukaryotic communities were analyzed by eDNA metabarcoding of the partial COI Leray-XT fragment. The results showed that eukaryotic communities inside the cages were significantly different from those in the outside environment, with communities inside the cages having higher diversity values and more indicator species associated with them. This is likely explained by the appearance of fouling species that colonize the artificial structures, but also by other species that are attracted to the cages by other means. Moreover, these effects were highly localized inside the cages, as the communities identified outside the cages, both North and South, had very similar eukaryotic composition at each point in time. Overall, the eukaryotic communities, both inside and outside the cages, showed similar temporal fluctuations through the summer months, with diversity peaks occurring at the end of July, beginning of September, and in the beginning of November, with the latter showing the highest Shannon diversity and richness values. Hence, our study suggests that seasonality, together with salmonid aquaculture, are the main drivers of eukaryotic community structure in surface waters surrounding the farm.

## Introduction

Human activities are one of the main threats to the stability of marine ecosystems, producing structural and functional changes in marine habitats that hamper the ecosystems’ capacity to provide goods and services (Halpern et al., 2008; Claudet and Fraschetti, 2010). Therefore, a proper assessment on the distribution and intensity of human activities, and the appraisal of their impacts on marine ecosystems, is of crucial importance for the sustainable use of the ocean biodiversity (Halpern et al., 2008, 2012). Among the variety of human stressors, aquaculture represents one of the main threats for coastal marine environments (De Silva, 2012) and their associated ecosystems (Sarà et al., 2011; Taranger et al., 2015), as it is a rapidly growing industry that contributes up to 46% of the total global fish output (FAO, 2018). Environmental impact assessments regulated by national and international directives (e.g., Marine Strategic Framework Directive, MSFD in the EU) are required to maintain a healthy trade-off between ecosystem services and exploitation and to protect, conserve and enhance marine ecosystems.

Aquaculture impacts on the natural environment are widely recognized and monitored (Holmer et al., 2005, 2008; Kalantzi and Karakassis, 2006). Impacts include the release of particulate matter into the environment, which usually leads to significant ecological changes, such as shifts in macrofaunal communities, decrease in species diversity or complete removal of native infauna (Holmer et al., 2008; Keeley et al., 2014; Stoeck et al., 2018a). Most monitoring programs focus on the benthic impacts, which are easier to measure than in the water column, and they traditionally include inventories of benthic macroinvertebrates and detection of presence/absence of specific species identified by morphological taxonomy, which are used as indicators of ecosystem health (Aylagas et al., 2014; Keeley et al., 2014). A variety of benthic indices, such as AZTI’s Marine Biotic Index (AMBI) (Borja et al., 2000), have been developed to classify the degree of impact of a certain area and prompt the consequent restoration measures (Diaz et al., 2004; Pinto et al., 2009). However, little is known about the small-scale changes occurring at the surface waters of aquaculture sites, where interactions with wild populations, spreading of diseases and release of parasites from farms are also of environmental concern (Holmer et al., 2008). Moreover, such effects are mainly subjected to distance from the aquaculture cages and water current direction and velocity (Hamoutene et al., 2016). This includes the need for knowledge about the hydrodynamic patterns within and around aquaculture cages to properly understand the spatial and temporal variability of the environmental parameters to perform the correct assessment of the environmental impact of a aquaculture site (Klebert et al., 2013; Gansel et al., 2014).

Environmental DNA (eDNA) metabarcoding has revolutionized the way in which biomonitoring is performed, from single-species detection to community studies and environmental impact assessments (Bohmann et al., 2014; Thomsen and Willerslev, 2015; Pawlowski et al., 2021). eDNA is the combined genetic material of trace and community DNA that can be extracted from an environmental sample, such as water (Rodriguez-Ezpeleta et al., 2021). Analysis of eDNA can overcome the difficulties associated with traditional monitoring techniques such as the correct identification of cryptic species, the need for taxonomic expertise, the lack of standardized samplings or the invasive nature of some survey techniques (Thomsen and Willerslev, 2015). Moreover, eDNA data allows biomonitoring to be performed at higher temporal and spatial scales than traditional surveys (Gibson et al., 2015). Several studies using eDNA metabarcoding have already been performed to characterize benthic macrofaunal responses to aquaculture pressures and introduce its use to develop a genetic based marine biotic index for benthos (gAMBI) (Aylagas et al., 2014; Lejzerowicz et al., 2015; Pawlowski et al., 2018; He et al., 2020). Moreover, the application of high-throughput sequencing has facilitated the potential use of bacterial communities as bioindicators of aquaculture impact, as they rapidly respond to environmental changes (Stoeck et al., 2018a; Borja, 2018; Verhoeven et al., 2018; Armstrong and Verhoeven, 2020).

The implementation of eDNA-based technologies in routine biomonitoring is still hampered by the lack of consensus on whether it should only be applied to conventional bioindicators or also include new taxonomy-free bioindicators (Pawlowski et al., 2021), which use environmental genomics based profiling on communities and independently generated ecological status or known disturbance gradients (Cordier et al., 2021). Examples of new bioindicators have been found when considering all the operational taxonomical unit (OTU) profiles along known impact gradients such as eutrophication (Apothéloz-Perret-Gentil et al., 2017), oil spills (Bik et al., 2012), or aquaculture sites (Stoeck et al., 2018a). Cordier et al. (2021) proposed four general implementation strategies of environmental genomics for monitoring which include DNA-based taxonomic identification of known taxa, *de novo* bioindicator analysis, structural community metrics and functional community metrics. The visual identification of known bioindicators is only possible for macrofaunal species found in the sediments, where the majority of surveys are performed (Aylagas et al., 2014; He et al., 2020). However, taxonomy-free discovery of new bioindicators in the water column in the vicinity of aquaculture sites is likely to occur when planktonic communities are analyzed with eDNA methods, considering hydrodynamics and temporal patterns (Lanzén et al., 2021).

In the present study we aimed to assess the dynamics of eukaryotic communities inside an aquaculture farm through time using eDNA metabarcoding and contrasted the community composition with that obtained for the surface waters surrounding the aquaculture farm. To accomplish this aim, we 1) compared eukaryotic communities from filtered surface water taken inside salmonid cages and from nearby points around the aquaculture facility, 2) evaluated the use of eDNA metabarcoding for impact assessment, and 3) assessed temporal variability, by taking biweekly samples over a 5-month period.

## Materials and methods

### Study site and water collection

This study took place at a commercial salmon aquaculture farm (“Uløybukt”, locality number: 10726, position: 69° 51.605′, 20° 42.838′) located in the southern part of Skervøy municipality, Troms, Northern Norway. The farm was located in Rotsundet in a coastal area (Supplementary Figure S1) and is characterised by a sub-Arctic climate and water regime. Although high latitude Norwegian coastal areas are defined as ice-free, they have strong seasonality due to high variability of light intensity throughout the year (Wiedmann et al., 2016). The sampling was performed every 2 week from 20th July 2017 to 7th November 2017), accounting for a total of nine sampling dates. The farm had a total of 10 circular net-pins (diameter 140 m, depth ca. 40 m) composed by PE-plastic and nets of nylon/polyester and the distance between the net-pins were approximately 40 m. The depth at the locality were 50–95 m and the farm were located at a bottom consisting of a mix of sand, stones, and mud. Atlantic salmon smolts were stocked in the farm in April/May 2017 and it was estimated that farm had 800.000–1.000.000 fish during the sampling. At the beginning of the sampling the fish were distributed in seven net pins, whereas they were distributed in up to nine net-pins at the end of the sampling due to increased biomass. The fish were fed, based on appetite, throughout the day. In this study we defined outside environment as control and collected these samples at a distance 200 m from the cages North and South of the aquaculture farm, whereas samples taken inside the cage were considered as the treatment. In each sampling event, 2.5 L of water were collected at 1.5 m depth using a Niskin bottle (model 1010-2.5 L, GeneralOceanics) from five different spatial replicate points (A–E) inside three cages (M1, M2, M3) containing fish. Moreover, 2.5 L of water were also collected in five spatial replicates (A–E) in the outside environment at fixed points (200 m distance assessed with a GPS chart plotter) at the northern (N) and southern (S) side of the middle of the salmon farm at 1.5 m depth. All sampling equipment was sterilized with 10% bleach solution and 70% EtOH before each sampling event and thoroughly rinsed with seawater from the sampling point area before each use. All samples were collected and processed while wearing newly donned protective equipment such as nitrile gloves to prevent risk of contamination between samples or from outside sources.

Upon collection of the seawater samples, a filtering station was set up on site, and each sample bag was filtered through a 0.22 μm Sterivex filter units (Merck KGaA, Darmstadt, Germany) using a sterile 50/60 ml syringe from BD Plastipak. A total of 0.5 L of water were pressed through each of the five filters from each location to ensure a standard volume between samples. After drying the filters by pumping air through them, the filters were placed in prelabeled sterile 50 ml Falcon tubes (Thermo Fisher Scientific, Waltham, MA, United States), and prelabeled bags for transport to UiT The Arctic University of Norway (UiT) and long-term storage at −80°C in an eDNA dedicated freezer. The syringes were changed, and operators’ hands were meticulously sterilized, between each sample using 5% bleach solution and a MilliQ Ultrapure deionized water rinse to limit contamination. A control blank was run on each sampling day to quantify contamination during the filtering process by filtering 0.5 L of the remaining MilliQ rinse water through a filter and drying the filter in the same manner as the previous samples.

### DNA extraction

The Sterivex filters used for water sampling underwent DNA extraction in over-pressured eDNA clean-labs using trace eDNA extraction protocols specifically designed to prevent contamination from all airborne DNA present within UiTs facilities or present on the lab user’s skin, hair, or breath. These protocols relied on vigilant care for cleanliness within and outside of the eDNA laboratories and avoidance of potential contaminant sources at UiT and personal life during the weeks of eDNA extraction lab use. Airborne DNA contamination risks were mitigated through use of a pressure positive eDNA extraction rooms and airlocked changing and sample preparation rooms. eDNA extraction protocols were meticulously followed for the modified use of DNEasy Blood and Tissue® (Qiagen, Hilden, Germany) kits. In short, a total of 500 μl of lysis buffer were added to each Sterivex filter, sealed with sterile caps at both ends, and incubated 24 h on a rotary wheel placed in a 56°C incubator oven to ensure full lysis of the particulates captured within the filter membrane. The lysed solution was then centrifuged out of the filter casing and into 2 ml Eppendorf tubes and the rest of the extraction followed the standard protocol recommended by the extraction kit handbook (Qiagen 2020). Subsequently each sample was eluted in 75 μl elution buffer, of which 20 μl was aliquoted for library preparation and sequencing.

### PCR amplification and sequencing

A multiplexing approach was used for sequencing the 320 samples on an Illumina MiSeq next-generation sequencer (Illumina, San Diego, CA, United States). The partial COI Leray-XT fragment (313 bp) was amplified using the mlCOIintF-XT/jgHCO2198 primer pair (Wangensteen et al., 2018). Samples included 19 PCR blanks as well as field blanks for each sampling event. 8-base tags were used to uniquely label each sample as in Wangensteen et al. (2018). PCR amplifications were conducted in 20 μl reactions containing 2 μl of DNA template, 10 μl of AmpliTaq Gold Master mix, 0.16 μl of Bovine Serum Albumin (20 μg/μl), 1 μl of each forward and reverse primer (5 μM) and 5.84 μl of H2O. The temperature profile was as follows: 95°C for 10 min; 35 cycles × (94°C/1 min, 45°C/1 min, 72°C/1 min); 72°C/5 min. Only one PCR replicate was run per sample. The success of PCR amplifications was checked by gel electrophoresis in 1% agarose and PCR products were then pooled together into two multiplex sample pools. MinElute PCR purification columns (Qiagen) were used to concentrate the pooled DNA and to remove fragments below 70 bp. Library preparation was performed with the NEXTflex PCR-free library preparation kit (BIOO Scientific) and the exact library concentration was measured in a qPCR machine (ThermoFisher), using the NEBNext Library Quant Kit (New England BioLabs). Finally, pools were sequenced along with 1% PhiX on an Illumina MiSeq platform using v3 chemistry (2 × 250 bp).

### Metabarcoding pipeline

The OBITools v1.01.22 software suite (Boyer et al., 2016) was used for the initial steps of the bioinformatic analyses. Paired-end reads were aligned using *illuminapairedend* and only sequences with alignment quality score > 40 were kept. Demultiplexing was done using the sample tags with *ngsfilter*, which also removed primer sequences. Aligned reads with length of 299–320 bp and without ambiguous positions were selected using *obigrep* and then dereplicated with *obiuniq.* Chimeric sequences were removed using the *uchime-denovo* algorithm implemented in vsearch v1.10.1 (Rognes et al., 2016). Clustering of sequences into molecular operational taxonomic units (MOTUs) was performed using SWARM 2.0 (Mahé et al., 2015) with a *d* value of 13 (Bakker et al., 2019). Taxonomic assignment of the representative sequence of each MOTU was done with the *ecotag* algorithm (Boyer et al., 2016) using a local database of Leray fragment sequences (available from https://github.com/uit-metabarcoding/DUFA). COI sequences for the MOTUs of interest (abundances > 0.5% of the total reads) were manually checked for better match by BLAST search against the NCBI GenBank and BOLD databases, and best IDs were changed to reflect a higher percent match if one was found. MOTU best IDs were then reassigned to an appropriate taxonomic rank based on percent match to the assigned species. Sequences assigned to bacteria or to the root of the Tree of Life, contamination of terrestrial origin, and MOTUs that were present in the control samples with more than 10% of their total read abundance were removed.

### Data analysis

Statistical analyses were performed in R version 3.1.3 (https://www.R-project.org/) with the *vegan* package [version 2.5–6; (Oksanen et al., 2019)] and graphic visualisations were done with *ggplot2* package (Wickham, 2016). Reads were first transformed to relative abundances to build a Bray-Curtis dissimilarity matrix, which was used to assess the variance in community composition using Permutational Multivariate Analyses of Variance (PERMANOVA). Samples were categorized as a function of *Type* (cage, outside), and *Date* (9 levels) and the univariate effects of these factors on the community composition were tested using *adonis* function with 999 permutations. Additionally, PERMDISP analysis (*betadisper* function) was performed for significant factors to determine if their effect was due to different multivariate mean or to different heterogeneity of the groups. Non-metric multidimensional scaling (nMDS) representation with Bray-Curtis dissimilarities was performed with the *metaMDS* function with 500 iterations. Shannon diversity and MOTU richness per sample were calculated in *vegan* (Oksanen, J. et al., 2019) after rarefaction to the lowest total number of reads per sample, to account for differences in sample sequencing depth. Then, two-way analysis of variance (ANOVA) was performed to detect significant differences between *Date* and *Type* in alpha diversity values.

An indicator species analysis (Dufrêne and Legendre, 1997) was performed in R using the *labdsv* package (Roberts, 2016) to detect potential associations of certain eukaryotic phyla to each type of environment (cage or outside). Those with Indval values > 0.5 (*p*-value < 0.05 in all cases) were selected as indicator phyla. The same analysis was performed to look for MOTUs associated to type of environment and to specific sampling dates. We retained the top 50 significant MOTUs with highest Indval values (in all cases these values are > 0.5 and with *p*-value < 0.05) as indicator MOTUs in each case.

Upset plots from the *UpSetR* package (Conway et al., 2017) were used to visualize the number of shared MOTUs between cage and outside environments for each sampling date and Treemaps from *treemapify* package (Wilkins, 2021) were created to visualize the overall eukaryotic composition of the sampling location considering read abundance and MOTU richness.

## Results

### The overall eukaryotic composition

After quality check, dereplication, chimera removal and manual filtering, we obtained a total of 6,985,791 reads assigned to 3471 eukaryotic MOTUs. After removal of MOTUs present in blank samples (58 MOTUs), and low sample reads (17 samples, <2000 reads), we obtained a final dataset of 6,847,336 reads assigned to 2984 eukaryotic MOTUs in 282 samples, which corresponded to 35 different Phyla. The average mean reads per sample was 22,915. Of the total MOTUs, 2906 were rare, with < 0.05% of the reads per MOTU, whereas only 17 MOTUs were considered abundant, with > 0.5% of the reads per MOTU.

**FIGURE 1:**
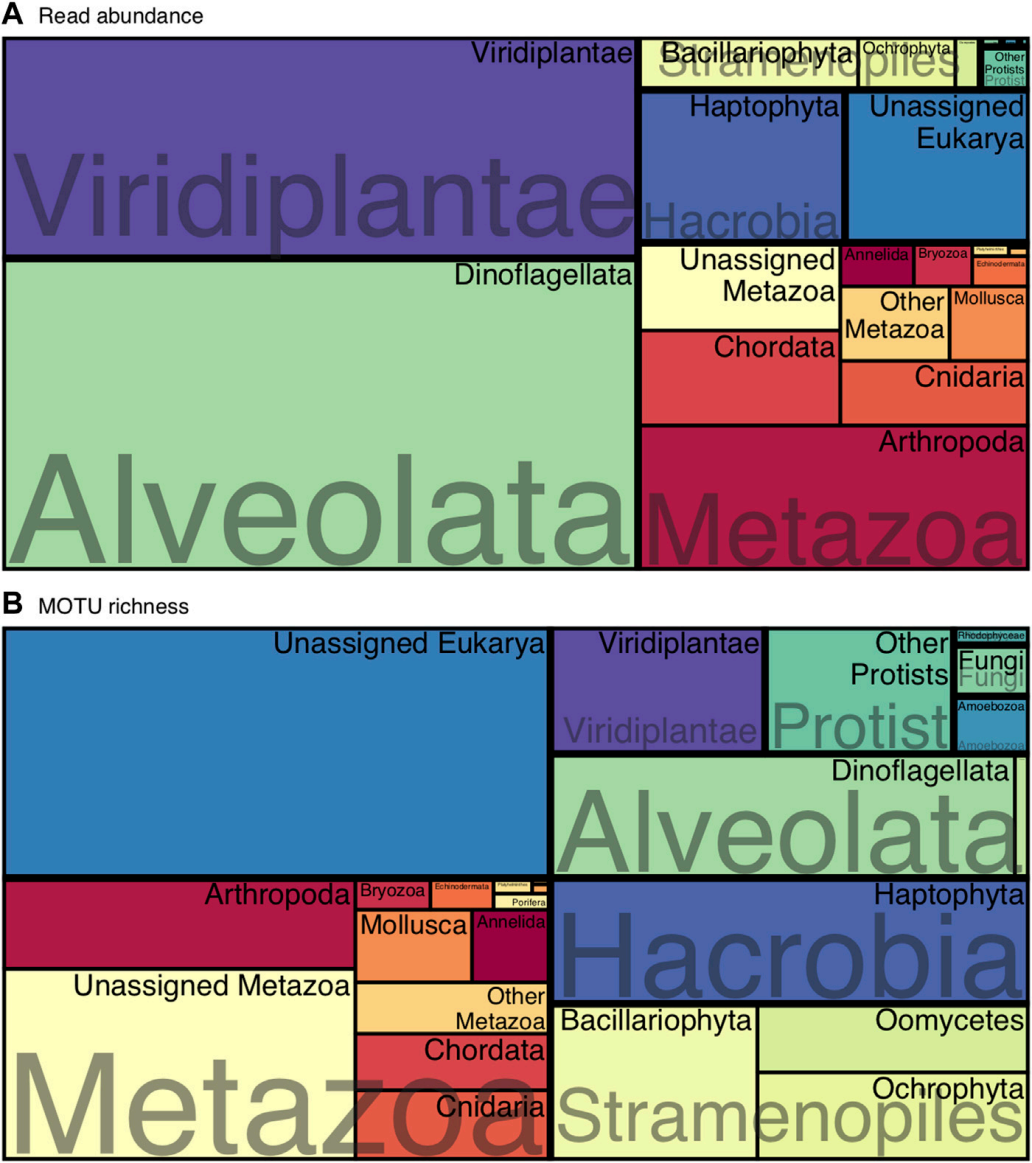
Treemap of the overall eukaryotic composition at phylum level considering **(A)** read abundances and **(B)** MOTU richness.

Dinoflagellates, Viridiplantae, Arthropoda and Haptophyta dominated the communities in terms of read abundance (Figure 1A), whereas the most MOTU-rich groups could not be classified at Phylum level and were represented by unclassified members of Metazoa or Eukarya (Figure 1B). However, those unclassified MOTUs represented a smaller proportion when considering their read abundance (Figure 1A). The following MOTU-rich phyla were Haptophyta, Dinoflagellata and Bacillariophyta (Figure 1B).

Within the most abundant MOTUs (Supplementary Table S1), an unassigned dinoflagellate (class Dinophyceae) was the most abundant in the whole dataset, followed by MOTUs classified as *Bathycoccus prasinos*, *Oithona similis*, and *Emiliania huxleyi.*

### Beta diversity

**FIGURE 2:**
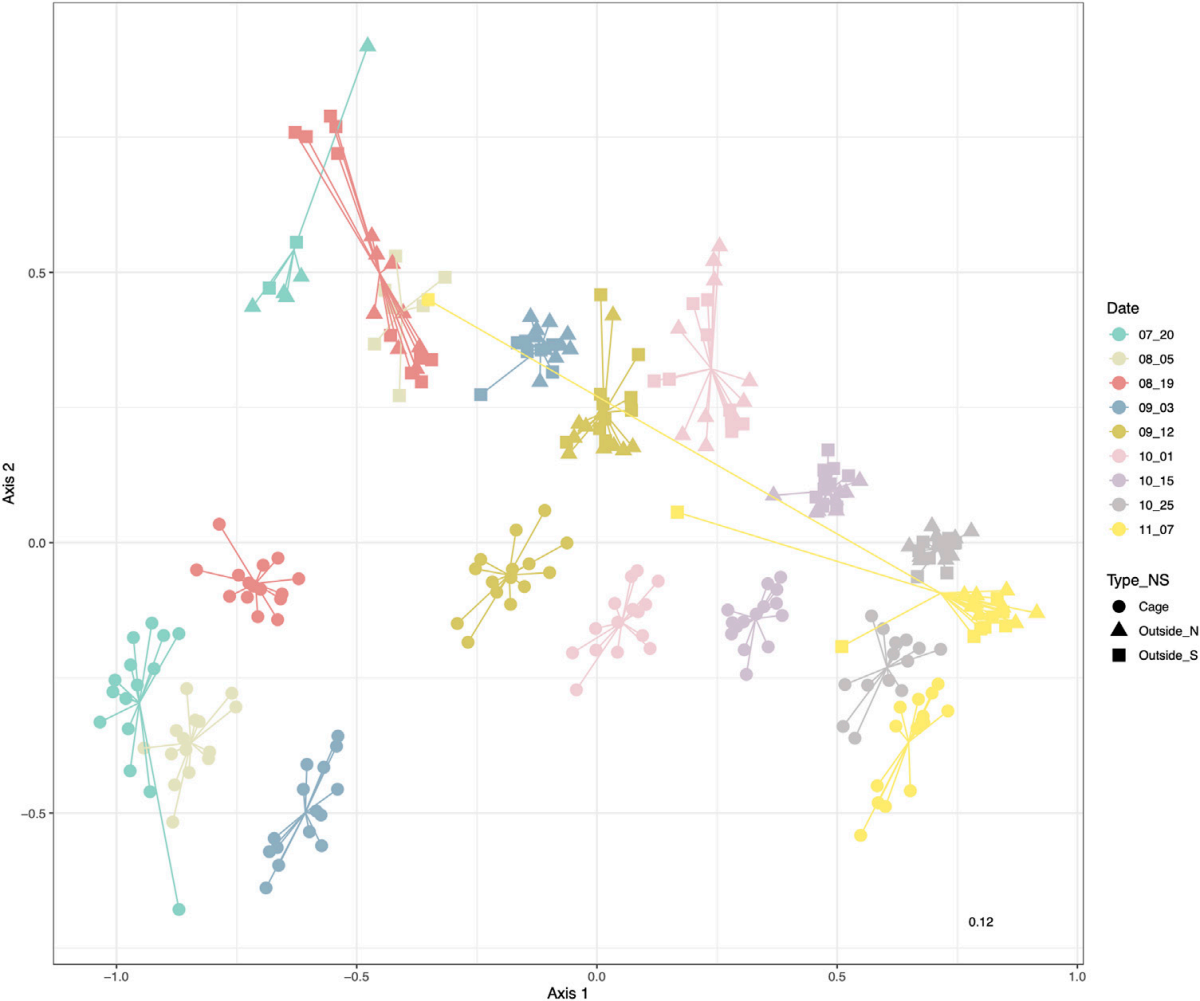
nMDS based on Bray-Curtis distances of sea water eukaryotic communities colored by sampling date. Shape corresponds to the environment inside the cages (circles) and North (triangle) or South (square) of the salmon facility.

The non-metric multidimensional scaling (nMDS) representation based on relative read abundance clearly showed that samples appeared ordered along the first dimension following the sampling date, while the second dimension separated samples from inside and outside the cages (Figure 2). Moreover, the nMDS ordination indicated that the eukaryotic communities from inside and outside the cages gradually differed less as the time went on and the sampling time approached the winter.

Initially we indicated that there were no significant differences between 1) the three cages and 2) between the North and South points of the outside environment (Supplementary Table S2), which were not significant (*p*-value > 0.05 respectively, Supplementary Tables S2.1, S2.2). Additionally, PERMANOVA with date and environment (inside and outside) as factors found significant differences and significant interaction between the factors (*p*-value < 0.001 respectively, Supplementary Table S2.3). PERMDISP analyses substantiated also differences in dispersion in these groups of samples (Supplementary Table S2). The *R* ^2^ value corresponding to the date factor (0.641) was greater than the one corresponding to environment type (0.079) or the interaction (0.061) (Supplementary Table S2.3).

### Community changes through time

**FIGURE 3:**
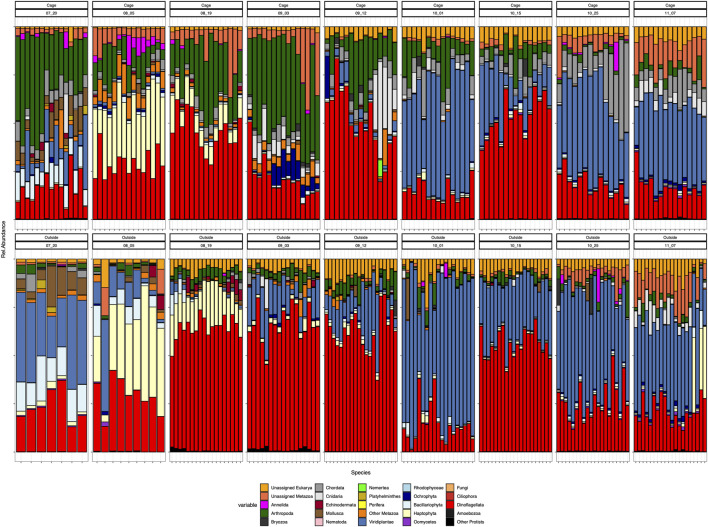
Composition at phylum level of all the samples. Cage samples on top and Outside samples in the bottom: ordered by sampling date Each color represents a different phylum.

In general terms we observe a shift in the predominance of dinoflagellates, especially during September, to Viridiplantae throughout October sampling dates (Figure 3). The dominance of dinoflagellates is more evident outside the cages, especially in the dates comprised between 19th August to 12th September, when they represent more than half of the total community abundance. However, inside the cages, this dominance is shared with other groups such as Arthropoda or unassigned members of Metazoa. Viridiplantae are almost absent inside the cage environment and in low abundance outside the cages during August and September and they clearly peak at the beginning of October, accounting for more than 50% of the relative abundance in both cage and outside-cage environments.

Eukaryotic communities were distinctly different between the environments inside and outside the cages in the first sampling dates (July) and they tend to have a more similar community composition by the last sampling date in November (Figure 3). During July, the cages are dominated by Arthropoda, which represent a small proportion in the outer environment, whereas Viridiplantae are the prevailing members of the outside-cages environment. Also noticeable is the presence of Annelida and Echinodermata inside the cages and their absence in the outer environment during July. Through time, both environments are mainly differentiated by the presence of certain phyla inside the cages, particularly Arthropoda the most evident during the first sampling dates and the increased abundance of Chordata and Cnidaria by the last samplings. Nevertheless, by the end of the sampling period, the eukaryotic composition of both environments resembles each other, dominated by Viridiplantae, Dinoflagellata, unassigned Eukarya and unassigned Metazoa, Arthropoda and Cnidaria, with the distinct presence of Chordata inside the cages due to the salmon presence.

Peaks of certain phyla at given dates are also noteworthy, such as the high abundance of Haptophyta during August in both environments, or the presence of Cnidaria and Ochrophyta inside the cages during September (Figure 3).

### Alpha diversity

**FIGURE 4:**
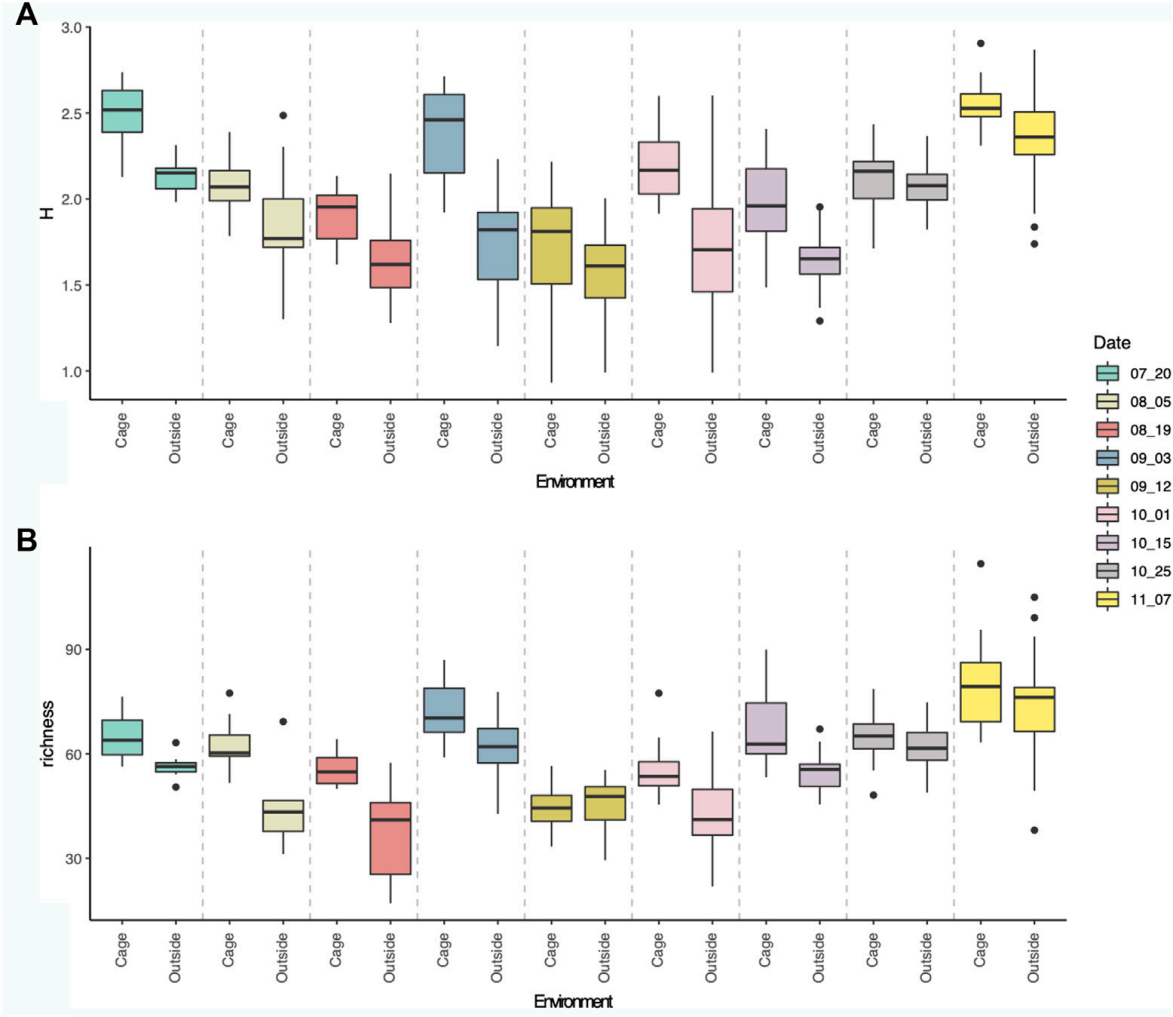
Boxplot showing Shannon diversity index **(A)** and Richness **(B)** of eukaryotic communities per sampling dates, inside (first) and outside (second) the cages.

Alpha diversity of eukaryotic communities associated to the aquaculture environment showed fluctuating values through time, with peaks diversity in July, beginning of September and highest values in November (Figure 4). The Shannon diversity and richness were always higher inside the cages than outside the cages, although both environments followed a similar fluctuating diversity pattern through time. This pattern showed a decrease in diversity after July diversity peak, followed by a peak at the beginning of September, reaching the lowest values in mid-September, whereafter an increasing trend was observed towards the highest diversity values in November (Figure 4).

Shannon diversity values ranged from 0.93 (12th September) to 2.90 (7th November), whereas richness values ranged from 17.13 (19th August) to 114.50 (7th November). Significant differences in Shannon diversity were observed between dates (Anova: F-value = 112,03, *p*-value < 0.0001), and environmental type (cage and outside, Anova: F-value = 33.75, *p*-value < 0.0001) and for the interaction of both factors (Anova: F-value = 4,05, *p*-value < 0.001). Similarly, significant differences were observed for richness values through time (Anova: F-value = 69,3, *p*-value < 0.0001) and environmental type (cage and outside, Anova: F-value = 43,9, *p*-value < 0.0001) and for the interaction of both factors (Anova: F-value = 3,65, *p*-value < 0.001). No significant differences were observed between different cages (M1, M2, M3) for Shannon diversity (Anova: F-value = 1.57, *p*-value > 0.05) or for richness values (Anova: F-value = 0.38, *p*-value > 0.05).

### Indicator species

**FIGURE 5:**
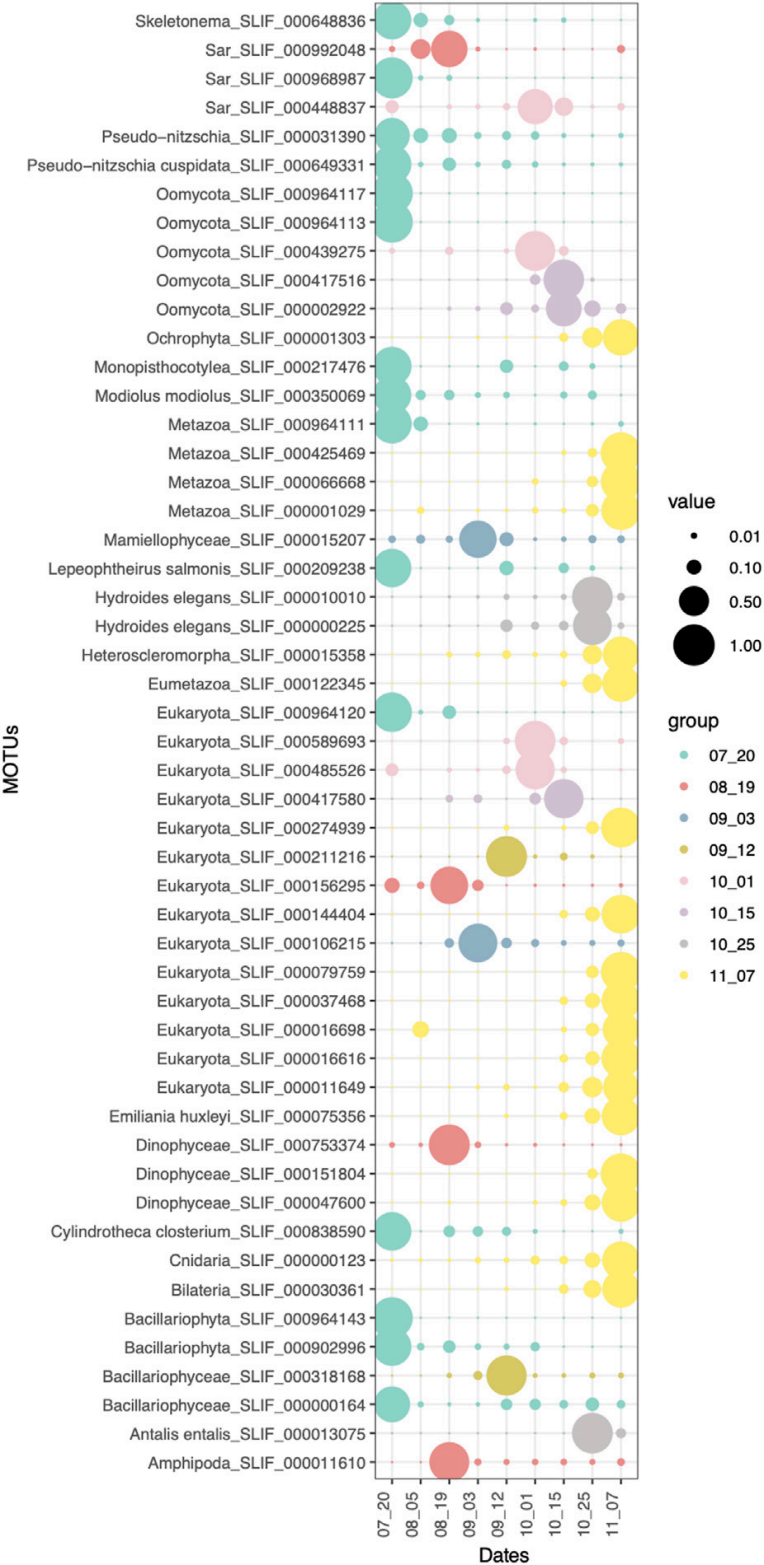
Indicator MOTUs (*p*-value < 0.05) of each sampling date. Size of the circles correspond to the relative abundances of each MOTU in each sample type.

Sampling time was the main variable affecting eukaryotic community structure (Figure 2). Therefore, eukaryotic communities found in the surface waters of aquaculture environment were differentiated between sampling dates, with certain taxonomic groups only present or enriched at given dates. The indicator species analysis for the *Date* factor shows which eukaryotic MOTUs are driving the main differences between sampling dates (Figure 5). First (20th July) and last (07th November) sampling have the highest number of indicator MOTUs, implying that they have the most differentiated communities, which is also coincident with the highest alpha diversity values for those dates (Figure 4). Relevant indicator MOTUs for July include MOTUs assigned to different species of the diatom *Pseudo-nitzschia*, the mussel *Modiolus modiolus*, the salmon louse *Lepeophtheirus salmonis*, and the diatom *Cylindrotheca closterium* (Figure 5)*.* The majority of indicator MOTUs for November were not taxonomically assigned to a specific species, except for a MOTU classified as the coccolithophore *Emiliania huxleyi.* Other indicator MOTUs that could be taxonomically classified at species level include the tube-forming worm *Hydroides elegans,* a harbor fouling invasive species, and the mollusk *Antalis entalis*, both highly abundant at the end of October (Figure 5).

**FIGURE 6:**
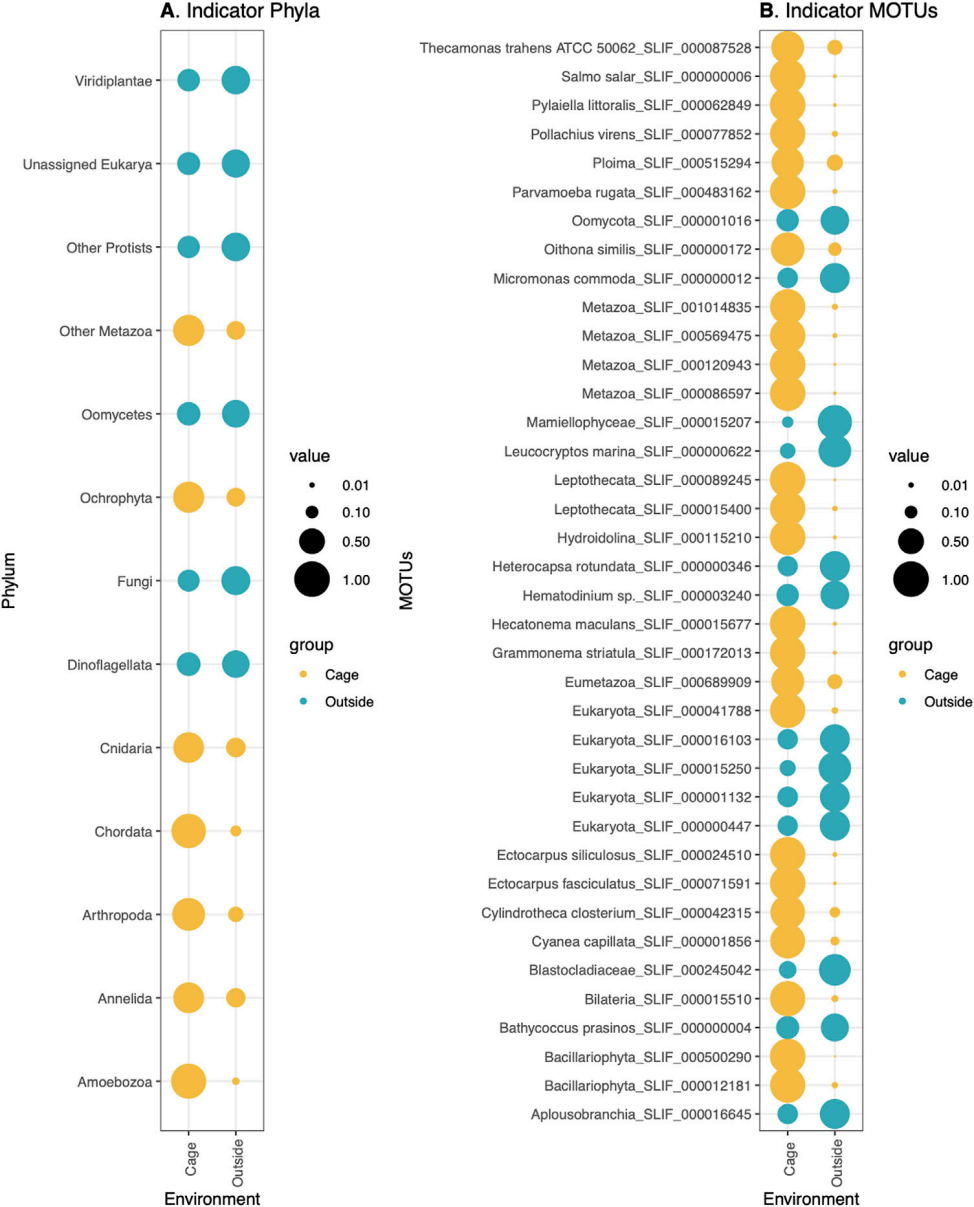
Indicator **(A)** phyla (*p*-value < 0.05) and **(B)** MOTUs (*p*-value < 0.05) of Cage (colored in yellow) or Outside environment (colored in Blue). Size of the circles correspond to the relative abundances of each phylum **(A)** or MOTU **(B)** in each sample type.

Significant differences in composition were also observed between the communities inside the aquaculture cages and the outer environment (Figure 2). The indicator species analysis at the phylum level (Indval > 0.5, *p*-value < 0.05) showed that Ochrophyta, Cnidaria, Chordata, Arthropoda, Annelida, and Amoebozoa were indicator phyla of the inside-cages environment, whereas Viridiplantae, Oomycetes, Fungi and Dinoflagellata were characteristic of the outer environment (Figure 6A). However, the differences in relative read abundance between cage and outside environments is much more evident for the indicator phyla of the cage environment, indicating that they have a more differentiated eukaryotic community (Figure 6A). Similarly, many MOTUs were found to be indicators of the cage environment, with highly different abundances compared to the outer cage environment (Figure 6B). Among those, relevant MOTUs belong to the fishes *Salmo salar* (Atlantic salmon) and *Pollachius virens* (Saithe), the brown algae *Pylaiella littoralis*, *Hecatonema maculans*, *Ectocarpus siliculosus*, and *Ectocarpus fasciculatus,* a rotifer belonging to the Ploima order, the himatismenid amoeba *Parvamoeba rugata*, the copepod *Oithona similis*, the diatom *Grammonema striatula*, the harmful diatom *Cylindrotheca closterium* and the lion’s mane jellyfish *Cyanea capillata* (Figure 6B)*.*

**FIGURE 7:**
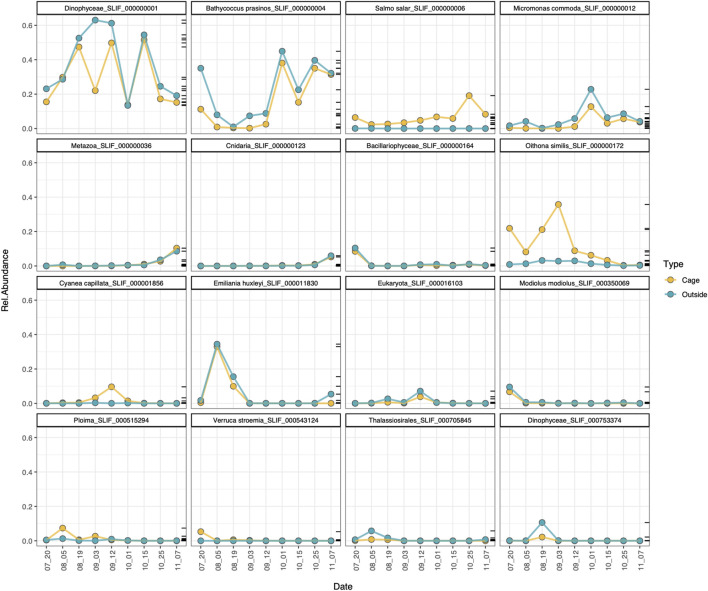
Temporal dynamics of the most abundant MOTUs (relab > 1%) in the cage (yellow) and outside (blue) environment.

The analysis of the most abundant MOTUs (relative abundance > 1%) confirms that most of the MOTUs follow the same temporal trend inside and outside the cages, peaking at the same dates, with the relevant exceptions of the MOTUs assigned to *Salmo salar*, *Oithona similis*, *Cyanea capillata* and the ploimid rotifer, which peak within the cages but are detected at low abundances in the outer environment (Figure 7). These results are consistent with the indicator species analysis at MOTU level for the environmental type, which found that those species were specifically associated to the cage environment (Figure 6B).

### Shared molecular operational taxonomic units between cage and outside environments through time

The Upset plot shows the number of shared MOTUs between the environment inside and outside the cages for each sampling date, as well as the total number of shared MOTUs for all the samples collected inside cages and for all the samples collected outside them through all sampling dates (Supplementary Figure S2). The results show that the number of shared MOTUs increase with time, being the last sampling date (7th November), the one with the highest number (671) of shared MOTUs between the cages and the outside environment. On the other hand, the initial sampling dates had fewer MOTUs in common, with values ranging from 261 to 387. Only 129 and 100 MOTUs were consistently found in all sampling dates inside and outside the cages, respectively (Supplementary Figure S2).

## Discussion

### Differentiated communities between inside and outside the cages

In this study, we have assessed the eukaryotic communities inside and outside salmonid aquaculture cages through a period of 5 months, using eDNA metabarcoding of water samples. Results show that the communities inside the cages significantly differ from the outside environment at distances of the order of 200 m, having eukaryotic communities with higher alpha diversity values and more indicator species associated with them. That is likely explained by the existence of artificial structures, such as the salmon cages, which allow for the settlement of new species. This colonization of organisms on submerged surfaces is known as biofouling, and it has several negative effects on farming equipment, water quality and fish health (Braithwaite and McEvoy, 2004; de Nys and Guenther, 2009; Guenther et al., 2010). Published information on fouling community in the Norwegian aquaculture industry is scarce and it has mostly been analyzed by visual census (Kvenseth, 1996; Guenther et al., 2009, 2010). Many of these fouling species have a rapid growth and present specific forms of attachment to the net which escape the naked-eye, hindering its complete removal during *in situ* washing of the nets (Guenther et al., 2009). While molecular methods are far from being a solution to the problem, they can offer a rapid and accurate assessment of the fouling communities, making it easier to identify the most problematic groups so that specific treatments can be applied. It is important to note that in the present study we did not specifically target the biofouling community, but rather the eukaryotic communities that are associated with the whole cage environment, giving a broader overview of the community changes. Among the indicator species that we found associated with the cages we can differentiate benthic and pelagic species. It is likely that the benthic species found, such as certain brown algae species, constitute the fouling community, while pelagic species represent other organisms associated with the cages by other means. This is the case, for example, for the saithe (*Pollachius virens*)*,* which swims close to the facilities and are common visitors and inhabitants inside the salmon cages (Otterå and Skilbrei, 2014). Species of rotifers and copepods were also found specifically associated with the cages, which may represent part of the salmon diet, although specific analysis of salmon feed components is required to confirm this hypothesis. Another species of interest is the lion’s mane jellyfish. It is likely that its polyp phase (scyphistoma) is associated with the cage structure and the peak in its eDNA is detected when strobilation occurs. In fact, jellyfish blooms have been widely reported in aquaculture facilities (Bosch-Belmar et al., 2017, 2019), causing significant gill damage to the Atlantic salmon (Baxter et al., 2011) and therefore, its monitoring is of great importance for preventing significant losses for aquaculture. *Pelagia noctiluca* and *Aurelia aurita* have been identified as the main agents causing mass fish deaths at salmon facilities in northern Europe (Doyle et al., 2008; Marcos-López et al., 2016). Our findings suggest that *Cyanea capillata* could also represent a threat for the aquaculture industry in Northern Norway.

In our indicator species analysis for dates, we also find relevant species that peak at specific times but without significant differences between the cages and the outside environment. Potential fouling species were found to have specific temporal peaks, such as the mussel *Modiolus modiolus* in July. Among those, it is important to note the detection of salmon lice, a major threat for the health of Atlantic salmon (Pike and Wadsworth, 1999). Lice counting and anti-lice treatments, such as the use of chemical bathing, cleaner fish, or mechanical removal, are routinely performed in the salmon facilities (Overton et al., 2019). Available data for salmon lice counts in our sampling facility show that it peaks in October (https://www.barentswatch.no/fiskehelse/fishhealthogram/10726/2017/44). However, our eDNA results show a significant abundance of salmon lice in July, coincident with the rise in the sea water temperature. This fact suggests that eDNA can be an efficient method to detect this parasite in the surface waters before it peaks inside the aquaculture cages later in the season, allowing for a better application of specific preventive anti-lice treatments at the right time. The higher alpha diversity values inside the cages can also be explained by the intermediate disturbance hypothesis, which states that species diversity is maximized at intermediate levels of disturbance because species that thrive at both early and late successional stages can coexist (Dial and Roughgarden, 1998). In that regard, the placement of aquaculture cages represents a disturbance to the natural ecosystem which allow for the settlement of r-selected species, such as seaweeds, which quickly colonize and dominate new environments. At the initial phases of disturbance, K-selected species that dominate the stable environments can thrive with the new colonizing r-selected species and thus, diversity is maximized. However, it is not only the colonization of new structures by itself that might imply the presence of more species, but also the changes in the primary productivity in the area, the rapid transfer of nutrients up the food web, or the attraction of wild fish communities to the floating structures (Holmer et al., 2008). Although during our 5 months sampling period, diversity inside the cages was always higher than outside the cages, a longer-term study following the whole production cycle is needed to assess if continued disturbance to the ecosystem leads to a final decrease in alpha diversity values. Several studies have reported decreases in alpha diversity metrics for certain taxonomic groups under anthropogenic impact (Pawlowski et al., 2014, 2016; Stoeck et al., 2018a, 2018b; Laroche et al., 2018), although increases in bacterial diversity and metabolic activity have also been detected in marine sediments (Galand et al., 2016; Pérez-Valera et al., 2017). Therefore, assumptions based on alpha diversity metrics should be considered carefully (Cordier et al., 2021), as higher diversity does not always imply a healthier ecosystem (Shade, 2017) and the introduction of invasive species is likely to occur with the placement of new structures into the natural environment.

Interestingly, the effect of the salmonid cages on the surface eukaryotic communities is highly localized, with the outside communities located North and South of the facility having highly similar eukaryotic compositions and differentiated from those inside the salmon cages. Such fine-scale differences in eukaryotic composition are likely to occur, as demonstrated by Antich et al. (2021) that found that only 7.5% of benthic MOTUs were retrieved in the water immediately adjacent to the benthos and that the number of shared MOTUs between water and benthos decreased as they moved apart from the benthic habitat (20 m.). In that sense, it is expectable to observe such differences in the eukaryotic communities only 200 m outside the aquaculture cages. However, it is relevant to point out that eDNA from the farm (i.e., trace levels of salmon DNA and other cage indicator species) can be still detected at these outer points, albeit at lower abundances.

A proper understanding on water movements is needed to understand the dynamics of eDNA around aquaculture facilities and should be considered in future studies assessing community metrics in the water column. Recent papers on hydrodynamic interactions on net panel and aquaculture fish cages (Klebert et al., 2013; Gansel et al., 2014) provide some insights into the flow dispersion around salmon facilities, being biological effects of fish, fish movements, and fouling, the major forces modulating the natural currents in these areas, influencing the redistribution of waste and nutrient-depleted water (Klebert et al., 2013). Indeed, the effect of fish swimming inside the cages generates currents that redirect the water flow (Chacon-Torres et al., 1988; Johansson et al., 2007; Klebert et al., 2013) with fish biomass, swimming behavior and schooling pattern of fish having differential effects on flow direction (Gansel et al., 2014). In the results of Gansel et al. (2014) the main fish biomass was found between 2 and 5 m depth, where fish were circling in the cage producing a rotational flow. Future studies that aim at the implementation of eDNA methods for aquaculture monitoring need a clear understanding of the hydrodynamics around salmon cages, considering different sampling depths and changes in the fish biomass, among others. Moreover, the time frame in which these studies are performed is also relevant, as we observe a homogenization of the outside and cage eukaryotic communities as the time goes on. At the initial sampling points, closer to the placement of the cages and the introduction of fish, eukaryotic composition between the two sampled environments is highly differentiated with low number of shared MOTUs between them. This can be explained by the creation of a completely new environment that produces a peak in diversity with the introduction of new species associated with the cages. Over time, communities inside and outside the cages tend to homogenize, which can indicate a localized effect of the farm to the outer environment. However, the temperature drop towards winter months can also explain the higher similarity between different environments as homogenization of eukaryotic communities occurs during winter months. A longer study following a whole annual cycle would be needed to confirm whether homogenization of both environments is occurring or not.

### General community composition and temporal variation

Although eukaryotic communities outside the cages significantly differ from those inside the cages, the temporal environmental patterns were the main driver of community composition for both environments, which have the same succession of the main phyla and similar diversity peaks through time. Dinoflagellates were the most abundant phylum from July to September and they were replaced by Viridiplantae from October to November. Dinoflagellates were also found to be the most abundant and diverse group in a study on protist diversity and seasonal dynamics in the Southern Norwegian coastal waters (Gran-Stadniczeñko et al., 2019) and they are considered to be one of the most important primary producers in the ocean (Not et al., 2012). Although the two most abundant dinoflagellate MOTUs could not be identified at species level due to gaps in COI reference databases, *Heterocapsa rotundata* was the third most abundant dinoflagellate MOTU. This species is a mixotrophic dinoflagellate that can ingest picoplankton and it is known to form large blooms in temperate estuaries during wet winters (Millette et al., 2017). It has been reported in a range of environments all over the word and tends to dominate the phytoplankton community for part of the year in some areas such as South Korea (Seong et al., 2006), Australia (Balzano et al., 2015), and Chesapeake Bay, United States (Millette et al., 2015). It is hypothesized that the mixotrophy of *H. rotundata* can give to this species an advantage over other phytoplankton species (Millette et al., 2017), allowing it to bloom under certain conditions. Among Chlorophyta (Viridiplantae), *Bathycoccus prasinos,* and the two picoflagellates *Micromonas commoda* and *Micromonas pusilla* were the most abundant MOTUs. These two latter species have recently been separated (Simon et al., 2017) and *M. pusilla* was shown to dominate the eukaryotic picoplankton in North Atlantic coastal and Arctic waters (Not et al., 2004). Similarly to our findings, *M. commoda* dominated the community in Oslofjorden (Gran-Stadniczeñko et al., 2019), and it is the second most dominant Chlorophyta species in Uløy bay. The third most abundant phylum was Arthropoda, with the small copepod *Oithona similis* being the most abundant MOTU. This result is consistent with the peak in abundance of this small copepod from June to December found in a Sub-Arctic fjord (Coguiec et al., 2021), which coincides with our sampling period. According to these authors, the autumn bloom (starting mid-September in Sub-Arctic waters) coincided with highest copepod diversity but also with a steep decline in zooplankton biomass driven by the decrease in abundance of the large *Calanus* species, which created a free niche in upper water layers that benefit small copepods, such as *Oithona similis* (Coguiec et al., 2021). Other Atlantic/boreal copepod species such as *C. helgolandicus*, *P. elongatus*, *T. longicornis*, and *P. elongatus*, were also detected in Uløy bay and found to be restricted to the Autumn bloom in a Sub-Arctic fjord, when strong south-west winds prevail in Tromsø area, forcing water of Atlantic origin into the fjord system (Coguiec et al., 2021). Finally, Haptophyta was the fourth most abundant phylum, with *Emiliania huxleyi* being the most abundant MOTU. This species was also found to be the most abundant haptophyte OTU in the Skagerrak strait, Southern Norway (Egge et al., 2015; Gran-Stadniczeñko et al., 2019).

Temporal variation in eukaryotic communities in terms of taxonomic composition and alpha diversity was also detected in our study, with peaks of certain species at specific sampling dates. Although communities inside the cages always presented higher diversity values than the outside environment, they both followed the same temporal fluctuations. It is well-known that plankton communities in Arctic and sub-Arctic marine waters present very strong seasonal changes in diversity and biomass due to the strong seasonality in solar radiation, snow and ice melt, river run-off and wind mixing, which produce stratification and mixing of water masses that govern nutrient availability (Coguiec et al., 2021). Year-round seasonal dynamics has been studied for zooplankton communities in a sub-Arctic fjord (Coguiec et al., 2021) and for protist diversity in Oslofjorden (Gran-Stadniczeñko et al., 2019), combining both molecular and morphological methods. Certain patterns found in those studies corresponding to our sampling period are comparable to the ones found in Uløy bay, such as the peak in diversity in November (Gran-Stadniczeñko et al., 2019) or the peak of *O. similis* in Autumn (Coguiec et al., 2021). Although our study did not attempt to study annual seasonality due to the restricted time frame, we did detect changes in community composition and dominant MOTUs through the sampling period that are equivalent to the spring-summer period (May–August) and the autumn-winter period (September–December) described for the zooplankton communities in a sub-Arctic fjord (Coguiec et al., 2021).

### Use of environmental DNA metabarcoding for eukaryotic monitoring in the water column

In the present study we have analyzed eukaryotic communities using eDNA metabarcoding of the COI fragment present in the surface waters inside and in the vicinity of salmonid aquaculture cages. To our knowledge, this is the first time that planktonic communities associated with aquaculture have been assessed by molecular methods, which provides a new perspective for aquaculture monitoring. Up to date, most of the molecular studies trying to monitor the impacts of aquaculture have focused on sediment samples, trying to standardize the traditionally used AMBI values (Aylagas et al., 2014, 2016) or generating new bioindicators (Cordier et al., 2017; Armstrong and Verhoeven, 2020; Frühe et al., 2020; He et al., 2020). However, molecular studies focusing on the planktonic communities have been largely neglected and the need for new protocols assessing the degree of change imposed by aquaculture on water quality and plankton dynamics have already been emphasized (Holmer et al., 2008). Our results show that eDNA methods can detect not only possible pathogens, but also members of the fouling communities and differential community composition between the cages and the outside environment. In the present paper we have used two of the proposed novel approaches to monitor ecosystems in Cordier et al. (2021); the taxonomy-free discovery of new bioindicators and structural community metrics. For the former one, we have used the indicator value approach to detect groups of MOTUs specifically associated with the cage environment, which significantly differ from the outside environment. Although promising, there are still several limitations to this approach, such as the gaps in the reference database that prevent the proper taxonomic identification of several indicator MOTUs, or the impossibility to assess the life stage of the indicator organisms. Specific assessment of species that may be identified by eDNA but are present in salmon feed is also an important step for future studies utilising this approach. In terms of community metrics, we have assessed differences in alpha diversity between the cages and the outside environment, which give a hint at the possible effects of aquaculture impacts. However, variation of alpha diversity alone is insufficient as a widely applicable indicator of disturbance (Cordier et al., 2021) and longer studies to evaluate diversity patterns under continued disturbance and detailed knowledge on the natural variability in the area are needed to extract significant conclusions. Finally, more detailed knowledge regarding the hydrodynamics around the salmon cages is of crucial importance to understand the eDNA dispersion flow in the water column and reveal the extent of the aquaculture impacts. Moreover, we acknowledge that our study only evaluated the eukaryotic communities in the surface waters at the close proximity of the salmon farm, but more effects are likely to be detected when addressing sediment communities or planktonic communities from further points.

## Conclusion

Analysis of eukaryotic communities inside and outside salmonid aquaculture cages through time revealed significant differences between both environments, with the cages having higher diversity values and more specific species associated with them. The placement of the cages creates structure that allows for the settlement of certain species that otherwise would not be found in the water column, explaining the higher diversity found within the salmon facilities. Interestingly, the effect of the cages on the eukaryotic communities of the surface waters surrounding the facilities was highly localized, with the communities located North and South of the cages having the same eukaryotic composition and being differentiated from the communities inside the cages. These results suggest that small-scale spatial changes in eukaryotic communities can be revealed by eDNA metabarcoding and provide additional rationale for the use of this method in impact assessment. Overall, the temporal pattern was the main driver of eukaryotic community structure, regardless of the environment studied (inside or outside the cages), with significant differences in alpha and beta diversity at given sampling times.

## Data Availability

The raw sequence reads for this study can be found in the Sequence Read Archive (SRA-NCBI) repository, Bioproject number: PRJNA839741.
