# Supplementary material for: Fine-scale differences in eukaryotic communities inside and outside salmon aquaculture cages revealed by eDNA metabarcoding
Source: Front Genet. 2022 Aug 26;13:957251. doi: 10.3389/fgene.2022.957251 (PMC9458982; doi:10.3389/fgene.2022.957251)
Supplement: Supplementary file 1 [file Table2.docx]

Table S2. PERMANOVA analyses for different factors explaining the variability in the eukaryotic communities are shown in the following tables. PERMDISP probability for homogeneity of dispersion are also shown with 999 permutations.

| *Factor* | *Df* | *SS* | *R2* | *F* | *P-value* | *PERMDISP* |
| --- | --- | --- | --- | --- | --- | --- |
| Cage | 2 | 0.2204 | 0.00854 | 0.56444 | 0.8452 | **< 0.05** |
| Residual | 131 | 25.5706 | 0.99146 |  |  |  |
| Total | 133 | 25.7909 | 1.000 |  |  |  |

S2.1. Cage (M1, M2, M3) only for cage samples

| *Factor* | *Df* | *SS* | *R2* | *F* | *P-value* | *PERMDISP* |
| --- | --- | --- | --- | --- | --- | --- |
| North/South | 1 | 0.2112 | 0.00958 | 1.412 | 0.2048 | > 0.05 |
| Residual | 146 | 21.8381 | 0.99042 |  |  |  |
| Total | 147 | 22.0493 | 1.000 |  |  |  |

S2.2. North and South only for Outside samples

| *Factor* | *Df* | *SS* | *R2* | *F* | *P-value* | *PERMDISP* |
| --- | --- | --- | --- | --- | --- | --- |
| Date | 8 | 33.869 | 0.64096 | 96.520 | **< 0.001** | **< 0.001** |
| Env. type | 1 | 4.152 | 0.07858 | 94.669 | **< 0.001** | **< 0.001** |
| Date:Env.type | 8 | 3.240 | 0.06131 | 9.233 | **< 0.001** |  |
| Residuals | 264 | 11.580 | 0.21914 |  |  |  |
| Total | 281 | 52.841 | 1.000 |  |  |  |

S2.3. Date, Environmental type (cage and outside) and interaction of both factors
