# Supplementary figures and images for: Fine-scale differences in eukaryotic communities inside and outside salmon aquaculture cages revealed by eDNA metabarcoding
Source: Front Genet. 2022 Aug 26;13:957251. doi: 10.3389/fgene.2022.957251 (PMC9458982; doi:10.3389/fgene.2022.957251)

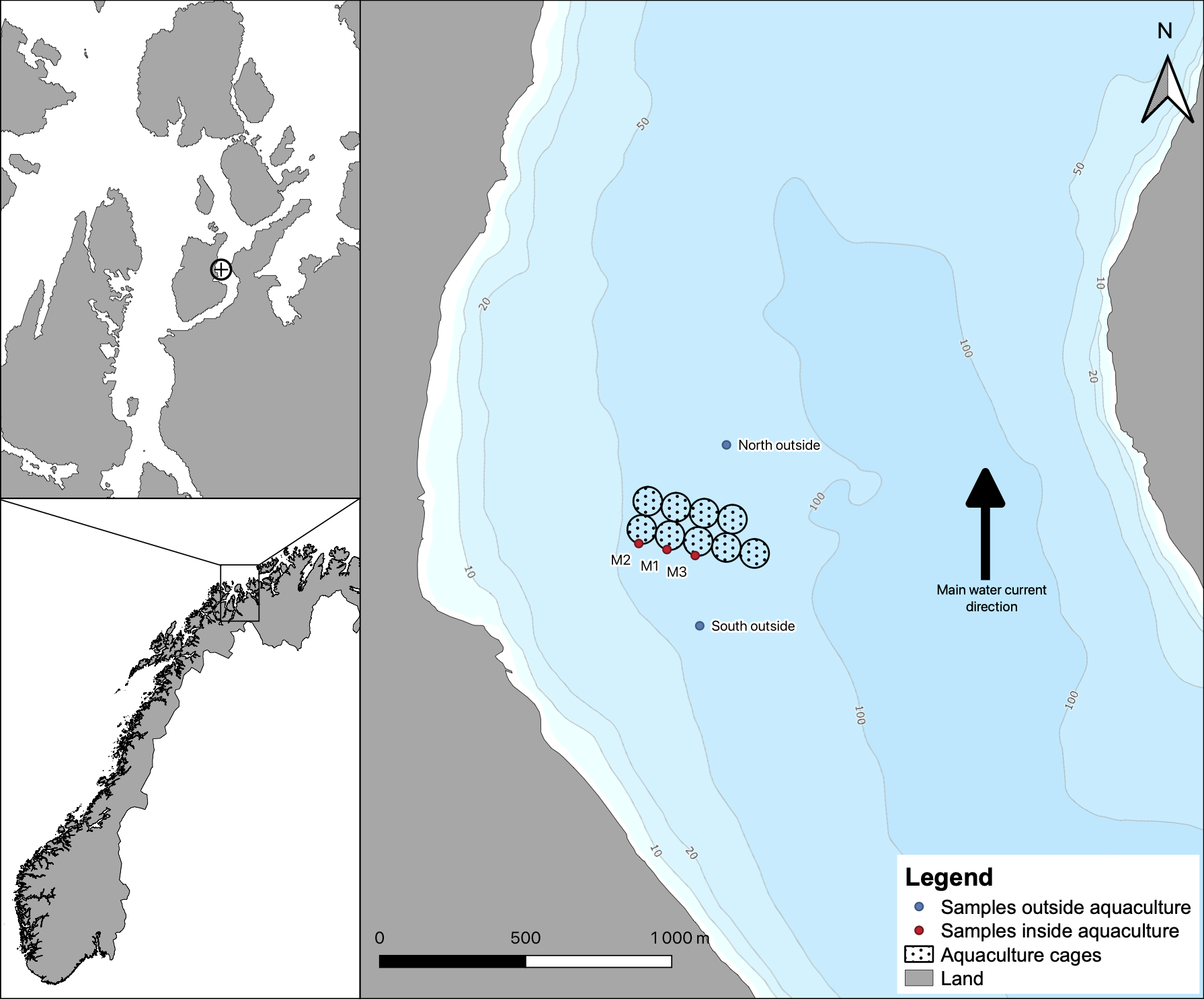

Supplement: Supplementary file 3 [file Image1.png]

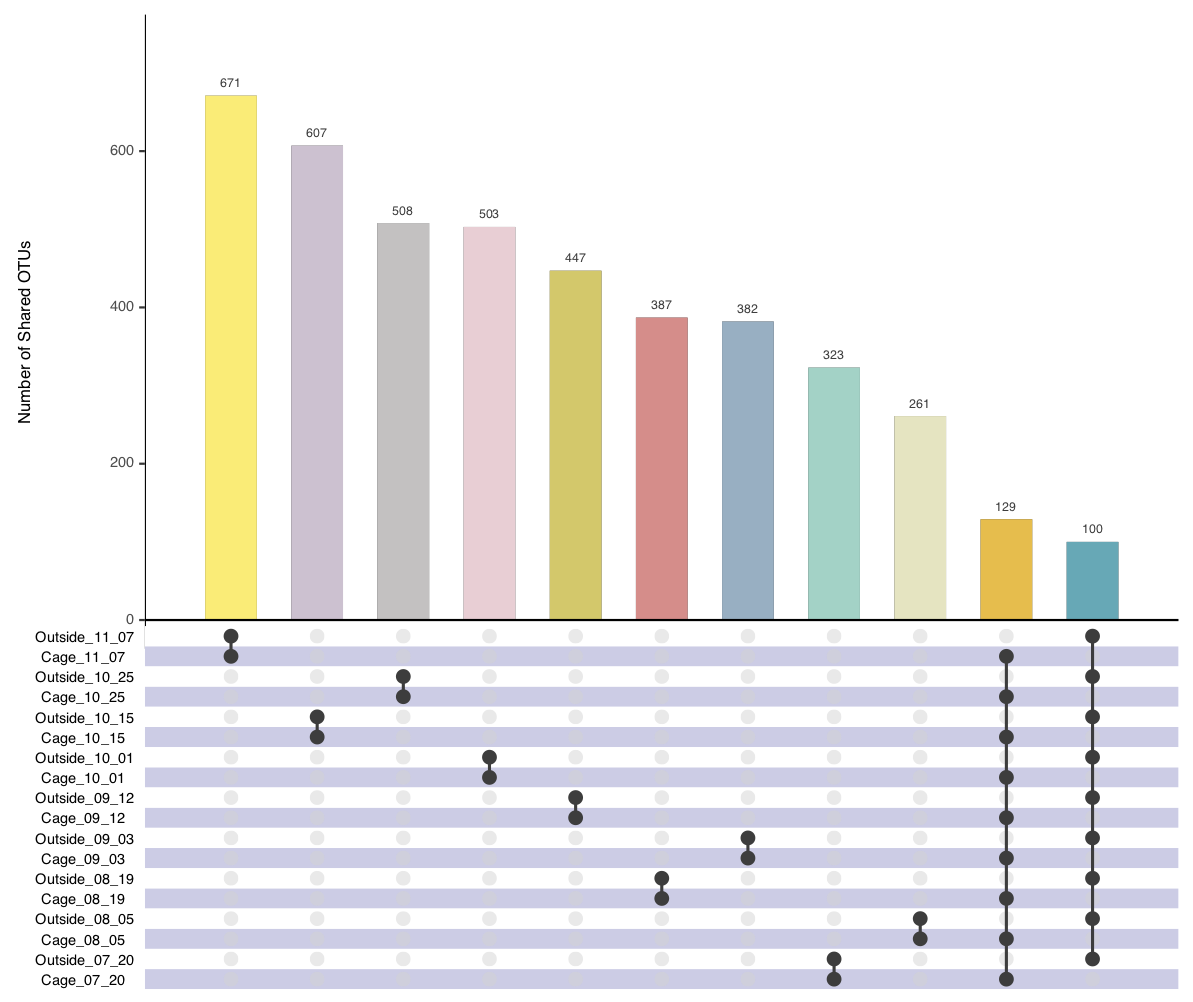

Supplement: Supplementary file 4 [file Image2.tiff]
